# Current Research Progress on ABHD5 in Cancers

**DOI:** 10.3390/cancers18040585

**Published:** 2026-02-10

**Authors:** Huazhong Cai, Hao Chen, Jiexing Ye, Zhesi Jin, Pan Huang

**Affiliations:** 1 Department of Emergency, Affiliated Hospital of Jiangsu University, Zhenjiang 212000, China; chenh723@163.com (H.C.); jinzhesi@126.com (Z.J.); 2 School of Medicine, Jiangsu University, Zhenjiang 212000, China; 3 School of Public Health, Anhui Mediecal University, Hefei 230000, China; 13655590443@163.com

**Keywords:** ABHD5, lipid metabolism, cancer, pathway

## Abstract

Lipid metabolism sits at the heart of tumor initiation, progression, metastasis, and resistance to chemotherapy. Against this background, the regulation of lipid flux has emerged as a fertile ground for anticancer strategies. Chanarin–Dorfman syndrome (CDS), a rare genetic disorder marked by massive lipid droplet accumulation, offers compelling human evidence that α/β-hydrolase domain-containing protein 5 (ABHD5) plays a central role in lipid droplet mobilization through the ATGL axis. This clinical insight has, perhaps unexpectedly, pushed ABHD5 into the spotlight of cancer research. ABHD5 does not behave uniformly across malignancies. In many solid tumors—such as lung, liver, and renal cell carcinoma—it restrains tumor growth. Yet in other settings, notably endometrial cancer, it appears to fuel malignant progression. Colorectal and prostate cancers occupy a more ambiguous middle ground, where ABHD5 can tip the balance in either direction depending on context. Mechanistically, ABHD5 influences lipid homeostasis and cell fate by intersecting with signaling pathways including AMPK/mTOR, AKT, and NF-κB, thereby shaping proliferation, invasion, apoptosis, immune evasion, and drug responsiveness. This review brings together experimental and clinical evidence to map the diverse, sometimes contradictory roles of ABHD5 in cancer. By tracing its context-dependent functions and molecular circuits, we also explore its emerging value as a diagnostic marker and a therapeutic target—one that demands nuance rather than blunt intervention.

## Simple Summary

ABHD5 is a key regulator of lipid metabolism, with context-dependent roles in cancer. It interacts with signaling pathways like AMPK/mTOR, AKT, and NF-κB, exerting either tumor-suppressive or oncogenic effects based on the tumor’s ecological and molecular context. This review synthesizes experimental and clinical evidence to clarify its multifaceted functions and explores its potential as a diagnostic marker and therapeutic target, emphasizing the need for precision strategies rather than blunt intervention.

## 1. Introduction

Cellular physiology is critically dependent on precise lipid metabolic regulation, which governs core biological activities. Abnormalities in this metabolic process have been demonstrated to be closely associated with the occurrence and development of tumors [1]. The α/β hydrolase domain (ABHD) family has emerged as a key regulator in lipid metabolism and tumor biology, attracting extensive research interest due to its pivotal functional roles [2]. Among the ABHD family members, ABHD5, also known as comparative gene identification-58 (CGI-58), stands out for its multifaceted and context-dependent roles in oncology, warranting a focused review [3,4]. The expression and activity of ABHD5 are subject to regulation by various factors, including nutritional status, hormone levels, and cellular stress. There is a demonstrable correlation between abnormalities in the function of the ABHD5 gene and a number of diseases. Mutations in the ABHD5 gene, which lead to a loss of protein function, are the established cause of CDS [5,6,7]. This disorder is characterized by the systemic accumulation of triglyceride (TG)-enriched lipid droplets (LDs) in tissue cells [8,9], underscoring ABHD5’s indispensable role in lipid mobilization. Beyond its critical role in systemic lipid homeostasis, ABHD5 also contributes to specialized physiological processes, such as the formation of the skin lipid barrier [10]. The functional significance of ABHD5 extends to pathophysiology, as its expression levels are significantly correlated with patient survival in various cancers (e.g., colorectal and prostate cancers) [11,12]. This broad impact likely stems from ABHD5’s involvement in fundamental cellular processes, including autophagy, cellular signaling, and membrane transport, highlighting its multifunctional nature in cellular physiology and disease. Notably, ABHD5 exhibits a striking functional duality in cancer that has become a core focus of related research. In most solid tumors (e.g., lung, liver, renal cell carcinoma), ABHD5 functions as a tumor suppressor by regulating lipid metabolism and downstream signaling pathways [13,14,15]; however, in specific contexts such as endometrial carcinoma, it acts as an oncogenic driver to promote tumor progression [16]. Colorectal and prostate cancers even display both tumor-suppressive and oncogenic effects of ABHD5, depending on the biological context [8,17,18,19]. These contradictions have left the field fragmented. For half a century, cancer research has been dominated by the linear reductionist “somatic mutation theory,” which frames cancer as genetically driven but fails to improve prognosis due to neglecting tumor cell-microenvironment crosstalk [20]. Emerging evidence instead positions cancer as an ecological and evolutionary process—a multidimensional spatiotemporal “unity of ecology and evolution” in a pathological ecosystem [20], where tumor cells act as “invasive species” and the tumor microenvironment (TME) forms the niche shaping progression via reciprocal interactions [20]. As a key regulator of lipid metabolism and signaling, ABHD5 is embedded in this ecological network, with its context-dependent dual roles reflecting adaptive responses to niche changes (e.g., nutrient availability, immune pressure, therapy). Therefore, this review aims to transcend fragmented narratives by integrating experimental and clinical evidence through the lens of cancer ecology, clarifying under what conditions ABHD5 suppresses tumors, when it promotes them, and the underlying mechanisms driving such functional divergence. This approach not only elucidates the context-dependent principles governing its pleiotropic effects but also provides an integrative theoretical framework for developing targeted therapies that address the systemic complexity of cancer.

## 2. The Structure and Function of ABHD5

The ABHD5 protein, comprising 349 amino acid residues, is transcribed from the CGI-58 gene located at chromosomal position 7p21.33 [21]. Understanding its unique structure is key to elucidating its central role as a master regulator of lipid metabolism, particularly its function in initiating lipolysis.

### 2.1. Structural Insights into ABHD5

This genetic locus contains eight protein-coding exons that encode for a member of the α/β hydrolase enzyme superfamily, exhibiting the conserved structural fold characteristic of this protein class [21]. The catalytic triad of typical lipases/esterases requires the GXSXG motif (where X can be any amino acid), with serine acting as the nucleophilic attacking residue. While ABHD5 shares sequences with other esterase/lipase/thioesterase subfamily members, the active serine residue in its GXSXG motif is replaced by aspartic acid. This results in the absence of intrinsic lipase activity. This unique molecular structure means that ABHD5 indirectly regulates lipolysis by activating lipases such as ATGL [2,22]. The ABHD5 protein architecture incorporates eight β-sheet segments, including a distinct antiparallel configuration in the second β-strand. These structural elements are interconnected through α-helical domains and cyclic motifs, collectively constituting the framework for the ligand-binding cavity [2,23]. The surface of ABHD5 identified more than 30 binding pockets, with the main binding pocket containing an average of 61 binding residues [24,25,26]. ABHD5 regulates lipid metabolism in a synergistic manner through two key binding pockets (α and θ) [27]. Pocket α comprises residues such as R299, G328 and D334, which directly activate the activity of the ATGL lipase [27]. Mutations (e.g., D334G/N) can significantly inhibit the function of lipolysis [27]. Pocket θ contains residues such as R116, G113 and D110, which target LDs, mediate interactions with Perilipin(PLIN) and activate ATGL lipase activity. Mutations (e.g., G113S and W93A) can disrupt localisation and interactions [27]. CDS-related mutations (e.g., H82R/H84K, E260K/E262K and S115G/S117G) are often found in these functional pockets. These mutations disrupt PLIN binding or cause charge reversal, resulting in lipid metabolism disorders [10]. The C-terminal region of ABHD5 harbors an evolutionarily preserved HXXXXD motif that demonstrates catalytic acyltransferase functionality. In contrast, the N-terminal segment contains a hydrophobic domain specialized for lipid binding, enabling molecular interactions with both lipid droplets and cellular membranes [23].

### 2.2. The Role of ABHD5 in Initiating Lipolysis

**Figure 1:**
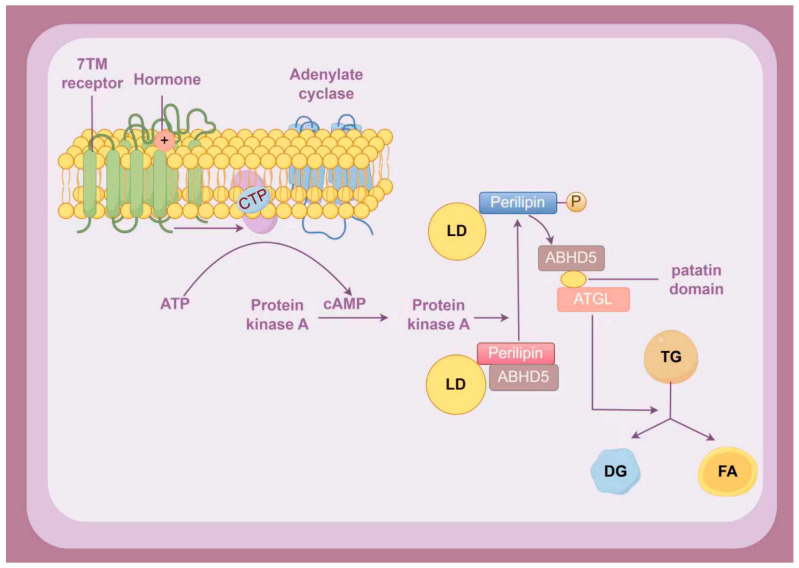
The molecular pathways through which ABHD5 modulates lipid metabolic processes. LD: lipid droplet. TG: triglyceride. FA: fatty acid. DG: diacylglycerol. CTP: Gαs-GTP (Gαs subunit bound to Guanosine Triphosphate). Hormone: including epinephrine, norepinephrine, and glucagon. Created by figdraw.com.

The ABHD5 protein structure comprises eight β-sheet segments (including a distinct antiparallel configuration in the second β-strand), interconnected by α-helical domains and loop motifs that collectively form the ligand-binding cavity framework [28]. The ligand-binding cavity framework is a 3D structural scaffold in proteins (e.g., ABHD5) formed by secondary structures like β-sheets, α-helices, and loops. It creates a specific pocket to bind ligands, enabling ligand-mediated protein function regulation [10]. ABHD5 has been identified as a key cofactor for adipose triglyceride lipase (ATGL). In this capacity, it binds directly to the patatin domain of ATGL, relieving its self-inhibitory conformation. This, in turn, activates the lipase activity of ATGL, catalyzing the conversion of TG into diacylglycerol (DG) and fatty acids (FA), thus initiating lipid metabolism [29,30]. Upon hormone-induced activation of the 7TM receptor-cAMP-PKA axis—specifically by lipolysis-promoting hormones including epinephrine, norepinephrine, and glucagon—phosphorylation of perilipin at Ser492/517 triggers ABHD5 release, enabling its binding to and activation of ATGL (Figure 1) [7,30].

## 3. ABHD5 and Tumors

Lipid metabolism disorders modulate tumor biology through multifaceted mechanisms, encompassing the induction of tumor-associated inflammation [31], stimulation of angiogenesis [32], regulation of stromal cell functionality, and facilitation of immune escape via remodeling of immune cell populations [33,34]. ABHD5, a pivotal regulator of lipid metabolism, exhibits tumor-suppressive functions in cancers but paradoxically promotes tumor progression in specific contexts. This functional dichotomy likely stems from its pleiotropic roles in lipid metabolism, autophagy, and cellular signaling pathways [35]. Furthermore, ABHD5 modulates tumor immune evasion and angiogenesis through regulation of tumor-associated macrophage polarization and functional reprogramming [36]. ABHD5 also plays an important role in regulating tumor cell apoptosis. It can inhibit tumor cell proliferation by activating the peroxisome proliferator-activated receptor alpha (PPARα) signaling pathway, and induce apoptosis by regulating the expression of Bcl-2 family proteins [37]. ABHD5 regulates autophagy in tumor cells, thereby modulating tumor cells survival and drug resistance [28]. The activation of ABHD5 can trigger an ineffective cycle of TG hydrolysis and synthesis, thereby activating AMPK and inhibiting mTORC1 signaling, ultimately suppressing tumors anabolism [38]. Analysis of the Kaplan–Meier Plotter database shows that high expression of ABHD5 is significantly associated with prolonged survival in patients with various cancers, such as lung cancer, gastric cancer, liver cancer, and ovarian cancer [17,39]. However, there are currently few systematic comparative studies on the specific mechanisms of action of ABHD5 in different types of cancer. To fill this gap, this paper summarizes the latest research evidence on the role and mechanisms of ABHD5 in various tumors, as shown in Table 1. It is important to note the distinction between correlative observations and causal mechanisms in the studies discussed. While clinical data robustly associate ABHD5 expression with various cancer outcomes, the specific molecular functions and causal roles attributed to ABHD5 are primarily derived from controlled experimental models.

### 3.1. The Context-Dependent Dual Roles of ABHD5 in Colorectal and Prostate Cancers

ABHD5 exhibits dual tumor-suppressive and oncogenic effects exclusively in colorectal cancer (CRC) and prostate cancer (PCa), with functional bias determined by cancer-specific metabolic states, tumor microenvironment (TME) components, and signaling pathway crosstalk. The contradiction between its roles arises from “cell-autonomous regulation” (tumor cell-intrinsic) versus “non-autonomous regulation” (TME-mediated) in CRC, and “lipid metabolic balance” in PCa—two distinct drivers of functional duality.

#### 3.1.1. Colorectal Cancer

In CRC, ABHD5 exhibits context-dependent functions, demonstrating tumor-suppressive as well as tumor-promoting effects.

In CRC, ABHD5 is frequently downregulated in clinical specimens, and its lower expression is correlated with increased histological aggressiveness. The mechanisms underlying its tumor-suppressive effects are multifaceted. Firstly, ABHD5 deficiency drives cancer stemness. Given that CRC largely originates from cancer stem cells, with c-Met being a key factor in maintaining them, the loss of ABHD5 triggers the nuclear translocation of the SET1A complex subunit DPY30 [46,47]. This activates SET1A, promoting YAP methylation and histone H3 lysine 4 trimethylation (H3K4me3) modification, which synergistically enhance YAP-driven c-Met expression and thereby sustain CRC stemness [8]. Beyond regulating stemness, ABHD5 deficiency also promotes metastasis by facilitating epithelial-mesenchymal transition (EMT). In CRC cells, the loss of ABHD5 inhibits the AMPK-p53 pathway, leading to enhanced aerobic glycolysis and consequently accelerating EMT [39]. This pro-tumorigenic impact is further evidenced by experimental models, where ABHD5 depletion induces oncogenic transformation and accelerates the malignant progression of colorectal cancer and the transformation of benign adenomas [39]. Consistently, adenomas with extensive ABHD5 deletions carry a higher risk of malignant transformation, and low ABHD5 levels are associated with metastatic disease (M1/Stage IV), postoperative recurrence, and poorer clinical outcomes [39].Furthermore, ABHD5 contributes to genomic stability and modulates the TME through autophagy regulation. It directly competes with caspase-3 (CASP3) for binding to BECN1, blocking BECN1 cleavage. This preserves autophagic activity, suppresses genomic instability and inflammation, and ultimately delays tumor progression [41,48,49]. Additionally, within the TME, ABHD5 expression is significantly lower in migratory tumor-associated macrophages (TAMs). In TAMs, ABHD5 suppresses the secretion of matrix metalloproteinases (MMPs) by inhibiting the NF-κB p65 pathway, thereby impairing cancer cell migration and metastasis [42].

Recent studies have further expanded the regulatory network of ABHD5 in colorectal cancer lip metabolism and tumor progression. Lu et al. identified phosphatidylinositol glycan anchor biosynthesis class K (PIGK) as a novel upstream regulator of ABHD5 in CRC [40]. Mechanistically, PIGK regulates lipophagy through the PIGK-ABHD5-PPARα signaling axis, where PIGK-induced ABHD5 upregulation modulates lipid droplet hydrolysis and energy metabolism, ultimately suppressing tumor growth [40]. This finding adds a new layer to the complex regulatory network of ABHD5 in CRC, highlighting that ABHD5-mediated lipophagy regulation is not only involved in intrinsic tumor cell signaling but also integrated with upstream regulators such as PIGK, which enriches our understanding of the context-dependent functional duality of ABHD5 in CRC.

These tumor-suppressive effects reflect ABHD5’s role as a metabolic checkpoint in the favorable ecological niche, preventing excessive proliferation of tumor cells through maintaining lipid and genomic homeostasis. From the perspective of cancer as a complex adaptive system, the tumor-suppressive functions of ABHD5—including inhibiting cancer stemness and EMT—represent its role in maintaining “systemic homeostasis” during the initial adaptive phase of colorectal cancer.

**Figure 2:**
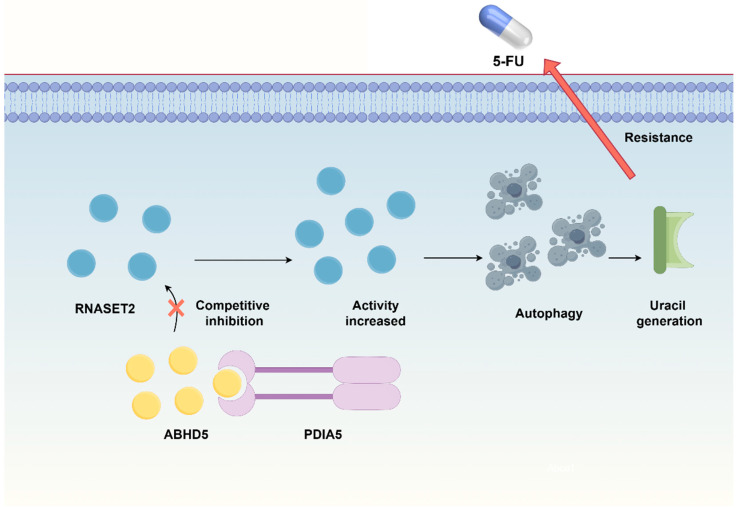
ABHD5-mediated uracil synthesis pathway conferring chemoresistance. Created by figdraw.com.

However, the role of ABHD5 in CRC is not exclusively tumor-suppressive, presenting a layer of complexity and controversy. Surprisingly, ABHD5 has been found to promote tumor progression in certain contexts by inhibiting spermidine dehydrogenase(SPDH)-dependent spermidine production. Since spermidine normally suppresses CRC cell proliferation, ABHD5-mediated reduction in spermidine alleviates this growth restriction, thereby facilitating tumor growth and metastasis [18]. Another critical aspect of its controversial role involves chemoresistance, particularly to the first-line drug 5-fluorouracil (5-FU) [50]. ABHD5 interacts with PDIA5 in lysosomes, as shown in Figure 2, competitively inhibiting the PDIA5-RNASET2 interaction. This preserves RNASET2 activity, promotes autophagy-dependent uracil generation, and ultimately reduces the chemosensitivity of CRC cells to 5-FU [43].

The dual nature of ABHD5 in CRC is not arbitrary but reflects the adaptive adjustments of tumor cells in response to dynamic changes in the niche—including treatment pressure. This dual role of ABHD5 in colorectal cancer is not accidental, but rather reflects the adaptive adjustments of tumor cells in response to dynamic changes in their surrounding environment. Clinically, for the same colorectal cancer patient, the function of ABHD5 may shift during treatment: at the initial stage of treatment, it inhibits the activity of cancer stem cells via the DPY30-YAP-c-Met signaling pathway and suppresses the EMT of tumor cells through the AMPK-p53 signaling pathway [8,39]. However, as treatment progresses, ABHD5 induces chemoresistance in tumor cells through the PDIA5-RNASET2-autophagy axis, thereby switching to an oncogenic role [43]. This dynamic functional switching underscores the limitations of traditional single-target therapies.

#### 3.1.2. Prostate Cancer

Prostate cancer (PCa) specimens demonstrate significant ABHD5 upregulation [19]. PCa presents a compelling case for the context-dependent functionality of ABHD5, as studies report both tumor-suppressive and tumor-promoting roles, often within the same cellular models. Reconciling these apparently contradictory findings requires a comparative analysis that considers the specific metabolic conditions, cellular dependencies, and experimental approaches employed.

**Figure 3:**
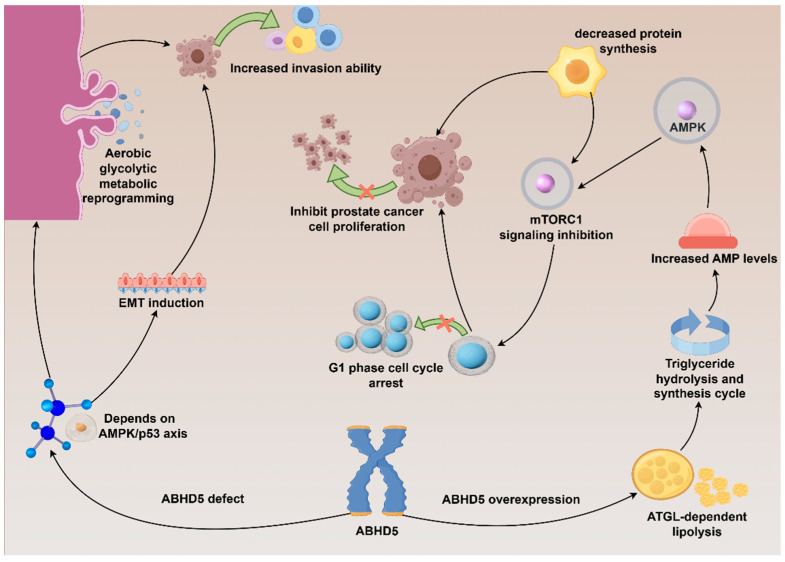
The bidirectional regulatory role of ABHD5 in prostate cancer cells. Created by figdraw.com.

On one hand, some evidence supports a tumor-suppressive role for ABHD5. According to research by Mitra et al., overexpression of ABHD5 triggers a cycle of triglyceride hydrolysis and synthesis through ATGL-dependent lipolysis [38]. This process increases AMP levels, activating AMPK, which in turn inhibits mTORC1 signaling, reduces protein synthesis, and suppresses prostate cancer cell proliferation. ABHD5 also induces G1-phase cell cycle arrest, further constraining proliferation [38]. Additional studies further indicate that ABHD5 deficiency has been shown to enhance the invasive potential of PCa cells by inducing EMT and promoting metabolic reprogramming towards aerobic glycolysis, effects that are dependent on an intact AMPK/p53 axis (Figure 3) [17]. Notably, recent research identifies ABHD5 as a novel suppressor of c-MYC-driven transcriptional programs in prostate cancer cells—its overexpression downregulates MYC target genes and reduces c-MYC protein levels time-dependently, while ABHD5 knockout elevates c-MYC expression, boosts cell proliferation and colony-forming capacity, and confers resistance to the c-MYC inhibitor 10058-F4. Critically, this tumor-suppressive function is independent of its canonical role as a PNPLA2 (ATGL) coactivator: PNPLA2 knockout fails to mimic ABHD5 loss’s effects on c-MYC and oncogenic phenotypes, and ABHD5 overexpression still suppresses c-MYC and inhibits proliferation in PNPLA2-deficient cells [44]. Moreover, pharmacological activation of ABHD5 with SR3420 robustly suppresses c-MYC time-dependently in castration-resistant PCa models (22Rv1, C4-2), highlighting its potential as a therapeutic target for MYC-driven prostate cancer and supporting non-canonical, ATGL-independent tumor-suppressive pathways that complement its known ATGL-dependent roles [44].

Conversely, several reports suggest that ABHD5 can be essential for PCa cell survival. Knockdown of ABHD5 in certain PCa models has been shown to reduce cell growth, promote significant lipid droplet accumulation, and induce apoptosis through activation of the AMPK/p70S6K pathway [19]. This implies that basal ABHD5 activity may be necessary to maintain lipid homeostasis and survival under specific conditions.

From a systemic perspective, the dual roles of ABHD5 in prostate cancer are closely linked to the “supply-demand status” of the tumor energy metabolic system. In nutrient-replete conditions, ABHD5-mediated lipolysis forms a “futile cycle” of triglyceride hydrolysis and synthesis, activating AMPK and inhibiting mTORC1 signaling to suppress tumor anabolism [38]. In contrast, under metabolic stress (e.g., hypoxia, nutrient limitation), ABHD5-driven lipolysis provides essential free fatty acids for energy production and membrane biosynthesis, becoming a critical survival strategy for the cancer system [19]. This aligns with the core viewpoint that tumor metabolism is a systemic adaptive process tailored to environmental constraints.

### 3.2. ABHD5 as a Tumor Suppressor

ABHD5 primarily functions as a tumor suppressor in most solid tumors, with a core common mechanism centered on regulating lipid metabolism-related signaling pathways (e.g., AMPK/mTOR, NF-κB) to inhibit tumor cell proliferation, metastasis, and immune evasion.

#### 3.2.1. Lung Cancer

Consistent downregulation of circ_cMras-ABHD5-ATGL signaling represents a pathognomonic feature in lung adenocarcinoma(LAC). Functional studies confirm this axis impedes cancer progression via NF-κB transcriptional inhibition. Mechanistically, ABHD5 serves as a downstream effector of circ_cMras and mediates its anti-tumor activity in LAC [45]. Notably, Chen et al. revealed that forced ABHD5 expression in the H1299 non-small cell lung cancer line markedly attenuates protein biosynthesis capacity, suggesting ABHD5-mediated metabolic inhibition as a potential anti-tumor mechanism [13]. Tripartite motif-containing 59 (TRIM59) is a new member of the tripartite motif family. As an oncogene, TRIM59 is significantly overexpressed in cancers, including lung and colorectal cancers [51,52,53,54]. ABHD5 physically interacts with TRIM59 to promote its ubiquitination and subsequent degradation. ABHD5 deficiency triggers metabolic reprogramming and NLRP3 inflammasome activation in macrophages, establishing a feedforward loop that accelerates lung cancer progression through IL-1β secretion [13].

The suppression of the NLRP3-IL-1β inflammatory loop by ABHD5 via promoting TRIM59 ubiquitination and degradation is essentially a regulation of the tumor immune microenvironment system [13]. ABHD5 deficiency disrupts the balance between tumor cells and macrophages, establishing a feedforward loop of “metabolic reprogramming-inflammation-tumor progression”—a classic example of systemic crosstalk in cancer. By blocking this inflammatory crosstalk, ABHD5 breaks the positive feedback cycle between the cancer cell compartment and the immune compartment, underscoring its potential as a systemic regulator of the tumor-immune interface.

#### 3.2.2. Liver Cancer

Hepatocellular carcinoma (HCC) specimens exhibit marked reduction in ABHD5 expression levels, with higher expression correlating with improved clinical outcomes, suggesting a potential tumor-suppressive role. As a key hydrolase domain-containing protein regulating hepatic lipid metabolism through lipolysis modulation and VLDL secretion, ABHD5 demonstrates potential as a therapeutic target for HCC. Notably, ABHD5 dysfunction is clinically associated with metabolic disorders including non-alcoholic steatohepatitis (NASH) and dyslipidemia, underscoring its central role in liver pathophysiology [55,56]. While extensive studies have established ABHD5’s pivotal role in hepatic lipid metabolism [57], its specific functions in HCC pathogenesis remained elusive until recent investigations elucidated its tumor-modulatory significance in liver cancer. Mechanistic studies indicate that ABHD5 not only promotes tumor cell apoptosis but also, as recently discovered, augments anti-tumor immunity through negative regulation of the immune checkpoint PD-L1—representing the first report of this immunomodulatory function [15]. Effective delivery of the ABHD5 gene can significantly improve the ability of T lymphocytes to kill tumors. Based on these findings, researchers developed an ABHD5 gene delivery system using injectable supramolecular hydrogels (PPA/CD). This system exhibits excellent gene loading capacity and sustained-release performance. It provides new strategies and clinical application prospects for gene therapy of liver cancer [15].

ABHD5’s negative regulation of PD-L1 represents its critical role in mediating “tumor-immune system” crosstalk [15], a function that extends beyond its classical role in lipid metabolism. Anti-tumor immunity is an integral part of the cancer system, and targeting immune-metabolic regulatory nodes can synergistically enhance therapeutic efficacy. The PPA/CD-mediated ABHD5 gene delivery system does not merely supplement a tumor-suppressive molecule, but repairs the disrupted “immune-metabolic regulatory system” in hepatocellular carcinoma, restoring the ability of T lymphocytes to kill tumor cells [15]. This systemic intervention strategy provides a paradigm for translating ABHD5’s systemic regulatory functions into clinical applications.

#### 3.2.3. Renal Cell Carcinoma

ABHD5 expression is downregulated in Renal cell carcinoma(RCC) tissues, with its mRNA reduction correlating with advanced pathological stages (including T stage) [14]. Functional studies suggest a potential mechanism, as ABHD5 overexpression suppresses tumor progression in experimental models via AMPK/mTOR pathway inhibition, leading to attenuated proliferation and metastasis [14]. FOX proteins are evolutionarily conserved transcription factors characterized by distinct functional domains [58]. These proteins play pivotal roles in developmental processes (e.g., embryogenesis) and cellular homeostasis, whereas their dysregulation contributes to tumor initiation and progression [59]. FOXC1 upregulates ABHD5 expression to modulate the AMPK/mTOR signaling pathway, thereby suppressing RCC cell proliferation and metastatic potential [14]. Conversely, ABHD5 knockdown abrogates the tumor-suppressive effects of FOXC1 overexpression, restoring RCC cell growth and invasion [14].

The FOXC1-ABHD5-AMPK/mTOR axis reflects a synergistic inhibitory network across the “transcriptional regulatory system” and “metabolic regulatory system” of renal cell carcinoma [14]. ABHD5 acts as an intermediate node that converts FOXC1-mediated transcriptional signals into metabolic inhibitory effects, blocking the transmission of proliferative signals in the cancer system [14]. This cross-system regulation highlights that ABHD5’s tumor-suppressive function is not limited to isolated pathway inhibition but involves integrating multiple systemic signals to reshape the tumor’s biological behavior. Effective cancer therapy requires targeting key nodes that mediate cross-system crosstalk, rather than single pathways, making ABHD5 a promising candidate for such strategies in renal cell carcinoma.

### 3.3. ABHD5 as an Oncogenic Driver

To date, ABHD5 has been identified as a tumor promoter primarily in endometrial carcinoma, with clear clinical relevance.

#### Endometrial Cancer

In endometrial carcinoma, ABHD5 functions as an oncogene, with its clinical relevance, mechanistic basis, and therapeutic implications well established. ABHD5 is upregulated in endometrial carcinoma and correlates with key clinicopathological parameters including patient age, histological grade, FIGO staging, lymphatic metastasis, and myometrial infiltration, showing particularly strong associations with advanced-stage disease (FIGO III) and lymph node dissemination [16]. More importantly, high ABHD5 expression independently predicts poorer overall survival, underscoring its value as a prognostic biomarker [60]. Functionally, ABHD5 acts as a potent driver of tumorigenesis. Knockdown of ABHD5 in high-expressing HEC-1A cells significantly suppresses proliferation, invasion, and glycolytic metabolism, whereas its overexpression in low-expressing Ishikawa cells reciprocally enhances these malignant phenotypes [16]. Mechanistically, the oncogenic effect of ABHD5 is critically dependent on the AKT signaling pathway. The key evidence is that the allosteric AKT inhibitor MK-2206 completely abrogates the tumor-promoting effects induced by ABHD5 overexpression [16].

Collectively, these findings position ABHD5 not only as a prognostic marker but also as a promising therapeutic target, with the ABHD5-AKT axis representing a novel vulnerability in endometrial carcinoma. The oncogenic effects of ABHD5 in endometrial cancer—promoting proliferation, invasion, and the Warburg effect via AKT pathway activation—represent its role in driving the “synergistic evolution” of the cancer system’s metabolic reprogramming and invasive capacity [16]. AKT pathway activation by ABHD5 reshapes the tumor energy metabolic system to favor aerobic glycolysis, while upregulating EMT-related proteins enhances tumor cells’ adaptability to the TME [16]. This dual regulation of metabolism and invasiveness by ABHD5 aligns with the systemic perspective of cancer progression—tumor cells acquire malignant phenotypes through coordinated remodeling of multiple biological processes. The ABHD5-AKT axis thus represents a critical systemic vulnerability in endometrial cancer, and targeting this axis may disrupt the coordinated evolution of the cancer system, providing a more effective therapeutic approach than single-target interventions.

### 3.4. ABHD5 Expression and Epigenetic Alterations in Cervical and Skin Tumors

Epigenetic alterations (methylation/deletion) of ABHD5 were identified in 21–44% of cervical cancer specimens, with significantly higher prevalence in squamous cell carcinoma (SCC) compared to adenocarcinoma (ADC). While these modifications potentially contribute to cervical carcinogenesis, their precise mechanistic involvement warrants further investigation [61]. Chen et al.’s immunohistochemical analysis revealed distinct ABHD5 staining patterns across skin tumors: scattered punctate/vesicular cytoplasmic localization in 83% of sebaceous carcinomas, dense perivesicular aggregation in all sebaceous adenomas, and complete absence of staining in clear cell basal cell carcinomas (BCC) [62]. These findings demonstrate that ABHD5’s distinctive expression profile effectively discriminates sebaceous gland neoplasms from non-sebaceous tumors, particularly clear cell BCC, highlighting its potential as a novel diagnostic biomarker for sebaceous gland carcinoma [62].

## 4. Conclusions

This review comprehensively synthesizes the multifaceted roles of ABHD5 in cancer, centering on its context-dependent functional duality and the underlying molecular mechanisms. As a pivotal regulatory node in lipid metabolism, ABHD5 interacts with key signaling pathways such as AMPK/mTOR, AKT, and NF-κB, exerting tumor-suppressive effects in most solid tumors (e.g., lung, liver, and renal cell carcinoma), while acting as an oncogenic driver in specific malignancies like endometrial cancer. Notably, in colorectal and prostate cancers, ABHD5 exhibits distinct dual roles—switching between tumor suppression and promotion—shaped by tumor-intrinsic metabolic states, tumor microenvironment (TME) components, signaling pathway crosstalk, and dynamic changes in the tumor ecological niche. Consistent with the perspective that cancer is a systemic disease driven by complex ecological and evolutionary processes, the functional versatility of ABHD5 highlights that the role (pro-oncogenic or tumor-suppressive) and biological function (driver, passenger, or neutral) of a single gene are not inherently fixed but are profoundly modulated by the environmental and ecological context in which cancer cells reside. For instance, in colorectal cancer, ABHD5 initially inhibits cancer stemness and epithelial-mesenchymal transition (EMT) through the DPY30-YAP-c-Met and AMPK-p53 axes, respectively, exerting tumor-suppressive effects. However, under treatment pressure (e.g., exposure to 5-fluorouracil), it interacts with PDIA5 to promote autophagic uracil production, reducing chemosensitivity and switching to an oncogenic role. Similarly, in prostate cancer, ABHD5 suppresses tumor anabolism via the ATGL-lipolysis-AMPK/mTORC1 pathway under nutrient-replete conditions but supports cancer cell survival by providing free fatty acids through lipolysis under metabolic stress (e.g., hypoxia or nutrient limitation). Such context-dependent functional switching reflects the adaptive strategies of tumor cells in response to dynamic changes in their ecological niche, underscoring the limitations of reducing cancer progression to individual genetic or molecular alterations alone.

From a clinical perspective, the systemic and context-dependent nature of ABHD5 poses both challenges and opportunities for therapeutic intervention. Blunt activation or inhibition of ABHD5 is inherently risky, as it may inadvertently abrogate its tumor-suppressive functions while potentiating its oncogenic effects. Instead, a precision medicine approach guided by the tumor’s unique ecological and molecular context is essential. For example, disrupting the ABHD5-PDIA5 interaction could reverse 5-fluorouracil resistance in colorectal cancer patients with chemo-resistant tumors, while ABHD5 gene delivery combined with PD-L1 inhibitors may enhance anti-tumor immunity in hepatocellular carcinoma by restoring the disrupted immune-metabolic regulatory network. Additionally, targeting downstream context-specific pathways (e.g., AMPK agonism in ABHD5-deficient tumors or AKT inhibition in ABHD5-overexpressing endometrial cancer) offers viable strategies to harness ABHD5’s regulatory potential without compromising its context-dependent beneficial effects.

Cancer progression is a systemic adaptive process involving intricate crosstalk between tumor cells, the TME, and the host’s metabolic and immune systems. Future research on ABHD5 should adopt a systems biology framework, integrating single-cell omics, spatial transcriptomics, and context-aware functional assays to decipher its cell-type-specific interactomes and signaling networks across diverse genetic, metabolic, and ecological backgrounds. This will enable a deeper understanding of how ABHD5 integrates into the cancer “metabolism-immune-TME” system and how its function is rewired during tumor evolution and treatment. Such insights are critical for developing biomarker-guided, system-oriented therapeutic strategies that account for the unique biological wiring of each tumor context—moving beyond single-target interventions to address the root causes of therapy resistance and malignant progression.

In summary, ABHD5 serves as a paradigmatic example of how a single gene contributes to cancer’s complexity through context-dependent regulation. Its functional duality not only enriches our understanding of the molecular and ecological basis of cancer but also provides a blueprint for developing more effective, personalized therapeutic approaches. By embracing a systemic perspective that acknowledges the dynamic interplay between genes, cells, and their environment, we can better leverage ABHD5’s regulatory potential to improve clinical outcomes for cancer patients.

## Figures and Tables

**Table 1:** The expression patterns and functional roles of ABHD5 across diverse tumor types. The mechanistic descriptions in this table are primarily based on evidence from functional studies.

Cancer	Expression	Role in Tumors	Mechanisms	Reference
Colorectal cancer	Low	Anti-tumor	Inhibits cancer stemness via DPY30-YAP-c-Met axis.	[8]
			PIGK upregulates ABHD5 to promote lipophagy via PIGK-ABHD5-PPARα axis, thereby inhibiting cell proliferation.	[40]
			Suppresses EMT by activating AMPK-p53 pathway.	[39]
			Preserves autophagy by competing with CASP3 for BECN1 binding.	[41]
			Inhibits TAM-mediated metastasis via NF-κB p65/MMP pathway.	[42]
		Pro-tumor	Promotes growth by inhibiting spermidine dehydrogenase.	[18]
			Reduces 5-FU sensitivity via PDIA5-RNASET2-autophagy axis.	[43]
Prostate cancer	High	Anti-tumor	Suppresses invasiveness via AMPK/p53-EMT axis.	[17]
			Suppresses c-MYC-driven transcriptional programs and proliferation in a PNPLA2-independent manner, sensitizing cells to c-MYC inhibitor.	[44]
			Inhibits proliferation via ATGL-lipolysis-AMPK/mTORC1 pathway (induces G1 arrest).	[38]
		Pro-tumor	Knockdown triggers lipid droplet accumulation-AMPK/p70S6K axis, inducing apoptosis (baseline expression supports cell survival)	[19]
Lung cancer	Low	Anti-tumor	Inhibits progression via circ_cMras-ABHD5-ATGL-NF-κB axis.	[45]
			Suppresses macrophage inflammation by promoting TRIM59 ubiquitination/degradation (blocks NLRP3-IL-1β loop).	[13]
Liver cancer	Low	Anti-tumor	Enhances anti-tumor immunity by negatively regulating PD-L1.	[15]
Renal cell carcinoma	Low	Anti-tumor	Suppresses proliferation and invasion via AMPK/mTOR axis.	[14]
Endometrial cancer	High	Pro-tumor	Promotes proliferation/invasion via AKT pathway activation.	[16]
			Enhances Warburg effect.	
			Upregulates EMT-related proteins.	

## Data Availability

No new data were created or analyzed in this study. Data sharing is not applicable to this article..
